# Progressive Compromise of Nouns and Action Verbs in Posterior Cortical Atrophy

**DOI:** 10.3389/fpsyg.2018.01345

**Published:** 2018-08-03

**Authors:** Brenda Steeb, Indira García-Cordero, Marjolein C. Huizing, Lucas Collazo, Geraldine Borovinsky, Jesica Ferrari, Macarena M. Cuitiño, Agustín Ibáñez, Lucas Sedeño, Adolfo M. García

**Affiliations:** ^1^Laboratory of Language Research (LILEN), Institute of Cognitive and Translational Neuroscience (INCYT), INECO Foundation, Favaloro University, Buenos Aires, Argentina; ^2^Laboratory of Experimental Psychology and Neuroscience (LPEN), Institute of Cognitive and Translational Neuroscience (INCYT), INECO Foundation, Favaloro University, Buenos Aires, Argentina; ^3^National Scientific and Technical Research Council (CONICET), Buenos Aires, Argentina; ^4^Department of Language Speech, Institute of Cognitive Neurology, Buenos Aires, Argentina; ^5^Faculty of Psychology, Favaloro University, Buenos Aires, Argentina; ^6^Faculty of Psychology, University of Buenos Aires, Buenos Aires, Argentina; ^7^Universidad Autónoma del Caribe, Barranquilla, Colombia; ^8^Center for Social and Cognitive Neuroscience, School of Psychology, Universidad Adolfo Ibáñez, Santiago de Chile, Chile; ^9^Centre of Excellence in Cognition and its Disorders, Australian Research Council, Sydney, NSW, Australia; ^10^Faculty of Education, National University of Cuyo, Mendoza, Argentina

**Keywords:** noun-verb dissociation, embodied cognition, neurodegeneration, posterior cortical atrophy, verbal fluency, MRI

## Abstract

Processing of nouns and action verbs can be differentially compromised following lesions to posterior and anterior/motor brain regions, respectively. However, little is known about how these deficits *progress* in the course of neurodegeneration. To address this issue, we assessed productive lexical skills in a patient with posterior cortical atrophy (PCA) at two different stages of his pathology. On both occasions, he underwent a structural brain imaging protocol and completed semantic fluency tasks requiring retrieval of animals (nouns) and actions (verbs). Imaging results were compared with those of controls via voxel-based morphometry (VBM), whereas fluency performance was compared to age-matched norms through Crawford’s *t*-tests. In the first assessment, the patient exhibited atrophy of more posterior regions supporting multimodal semantics (medial temporal and lingual gyri), together with a selective deficit in noun fluency. Then, by the second assessment, the patient’s atrophy had progressed mainly toward fronto-motor regions (rolandic operculum, inferior and superior frontal gyri) and subcortical motor hubs (cerebellum, thalamus), and his fluency impairments had extended to action verbs. These results offer unprecedented evidence of the specificity of the pathways related to noun and action-verb impairments in the course of neurodegeneration, highlighting the latter’s critical dependence on damage to regions supporting motor functions, as opposed to multimodal semantic processes.

## Introduction

Abundant neuropsychological evidence supports a dissociation between nouns and verbs – particularly, action verbs (Damasio and Tranel, 1993; Bookheimer, 2002; Chiarello et al., 2002; Cappa and Perani, 2003; Vigliocco et al., 2011). Whereas the former are typically compromised upon damage to posterior (mainly temporo-parietal) areas, the latter tend to become differentially impaired following lesions to frontal and subcortical motor regions (Pulvermüller et al., 1999; Shapiro and Caramazza, 2003; Luzzatti et al., 2006; Bocanegra et al., 2017). Far from being a random anatomo-clinical coincidence, this pattern has been sensibly interpreted from an embodied cognition framework (García and Ibáñez, 2016a, 2018; Birba et al., 2017).

This account highlights that nouns denote individuated, atemporal entities (such as objects or animals), typically identified by the collection of their sensory (e.g., visual, spatial, color, tactile) properties (Black and Chiat, 2003). Such a multimodal integration would be critically afforded by anterior and superior temporal as well as infero-parietal regions, given their well-established role as convergence hubs (Bookheimer, 2002; Patterson et al., 2007; Moseley and Pulvermüller, 2014; Ralph et al., 2017). Distinct noun-processing deficits can thus be logically predicted following temporo-parietal damage.

Instead, action verbs explicitly foreground bodily movements, sometimes involving the same effectors, and their identification hinges on recognizing the motion patterns they imply (García and Ibáñez, 2016a,b). Processing of these units would critically hinge on reactivations of the circuits which subserve the programming and execution of those movements, as systematically detected in frontal and subcortical motor regions (Moseley and Pulvermüller, 2014; García and Ibáñez, 2016a; Horoufchin et al., 2018; Pulvermüller, 2018). Indeed, lesions or atrophy of these areas entail selective or differential action-verb deficits (Arévalo et al., 2012; Cardona et al., 2013, 2014; Birba et al., 2017), arguably reflecting the disruption of embodied mechanisms (Birba et al., 2017; García and Ibáñez, 2018). Beyond its theoretical implications, this systematic link represents a promising avenue to develop early biomarkers across a range of brain diseases.

However, clean-cut dissociations between verbs and nouns are not always observed. For instance, difficulties in processing action verbs and other verb classes have been observed upon damage to the left ventral supramarginal gyrus (Meteyard et al., 2013) and other parietal and occipito-temporal regions (Taylor et al., 2017). On the other hand, noun impairments have been reported alongside verb disturbances following frontostriatal damage in Parkinson’s disease (Bocanegra et al., 2017) and frontal damage in non-fluent primary progressive aphasia (Marcotte et al., 2014). While this might seem to jeopardize de validity of the double dissociation originally postulated, a notably underexploited methodological strategy might shed light on the issue.

As it happens, besides the high variability of brain damage across patients (Crutch et al., 2012, 2017), these inconsistencies partly reflect the almost exclusive use of synchronic, one-time assessments, which preclude within-subject comparisons of performance as brain damage extends from one region to the next. A potentially useful way to overcome such limitations is to observe the progression of noun and action-verb processing deficits *in the course of neurodegeneration*. More particularly, given that nouns are easier to process than verbs (Goodglass and Geschwind, 1976; Zingeser and Berndt, 1990; Berndt et al., 1997; Thompson, 2003; Tyler et al., 2004; Mätzig et al., 2009; Shebani et al., 2017), a physiopathological model characterized by initial posterior damage progressively extending to frontal and subcortical motor hubs would offer a relevant testing ground. Such a pattern can be found in posterior cortical atrophy (PCA).

PCA is a syndrome characterized by posterior brain atrophy, a progressive decline in visual processing, and other higher-order impairments (Crutch et al., 2017), such as visual agnosia, disorientation, ocular fixation deficits, ataxia, anomia, agraphia, acalculia, and transcortical sensory aphasia (Tang-Wai et al., 2004; McMonagle et al., 2006). However, memory and language are relatively spared in early stages. The majority of PCA cases are attributable to an underlying disease, most commonly Alzheimer’s (Seguin et al., 2011).

Imaging studies have associated the syndrome with preferential atrophy of the occipital lobes (Kas et al., 2011; Panegyres et al., 2017), including the primary visual cortex and visual association areas (Schmidtke et al., 2005; Whitwell et al., 2007a; Migliaccio et al., 2009; Lehmann et al., 2011), posterior temporal regions (Schmidtke et al., 2005; Whitwell et al., 2007a; Migliaccio et al., 2009; Lehmann et al., 2011), and posterior parietal regions (Whitwell et al., 2007a) –especially the superior parietal cortex (Migliaccio et al., 2009; Lehmann et al., 2011). Predominantly right hippocampal atrophy has also been reported in PCA patients, with comparatively minor gray matter loss in striatal, insular, and inferior frontal regions (Whitwell et al., 2007a; Migliaccio et al., 2009). Importantly, as degeneration progresses, extended temporo-parietal damage is accompanied by atrophy of frontal and motor hubs (Lehmann et al., 2012; Ryan et al., 2014).

Against this background, we assessed the retrieval of animals (nouns) and actions (verbs) in a patient with PCA on two successive occasions: first, in an initial evaluation (Eval-1) at age 71, when atrophy was mostly confined to posterior regions supporting multimodal semantics; and then, in a follow-up evaluation (Eval-2) at age 73, when atrophy had progressed mainly toward fronto-motor regions. Imaging results were compared with those of controls via voxel-based morphometry (VBM), whereas fluency performance was compared to education- and age-matched norms through single-case statistics (see section “Fluency Performance Analysis”). We predicted that, if action verbs are critically grounded in motor regions, they should be selectively spared when atrophy is mainly posterior, despite their higher processing demands. Then, as neurodegeneration reaches frontal and subcortical motor hubs, deficits in this category should become evident and comparable to those in noun processing. This way, we aimed to assess the action-verb/noun dissociation in an overlooked though critical model of progressive neurodegeneration.

## Background

We present the case of a right-handed, 73-year-old man with PCA who reported no previous history of psychiatric or neurological disorders. He completed high school education, entered law school, and dropped out a few years later. He then started a gypsum company with a partner, in which he worked until retirement.

In 2006, at the age of 64, the patient consulted the Center for Memory Studies at the Institute of Cognitive Neurology, reporting non-progressive memory and attention deficits, episodes of spatial disorientation, and difficulty with spatial location of objects, recognition of known places, and reading –although ophthalmological examination indicated normal eye function.

An initial neuropsychological evaluation revealed impairments in tasks taxing visuoperceptual and visuospatial functions, such as localization of stimuli in space, visual memory, and visual attention. Auditory attention was normal and verbal memory tasks revealed difficulties in immediate paragraph recall with preserved word recall. In terms of linguistic abilities, the patient scored within normal ranges in tasks assessing naming, comprehension of complex grammatical structures, and phonological verbal fluency. However, the patient showed letter-by-letter reading. The assessment of executive functions indicated normal performance in tasks requiring motor series programming, responses to conflicting instructions, and verbal inhibitory control. Conversely, motor inhibitory control, working memory, and cognitive flexibility were slightly impaired. Finally, an initial brain MRI showed no evidence of neoplastic, vascular or inflammatory disease, but it revealed atrophy in the posterior parietal cortex, the post-central gyrus, and the superior parietal lobule.

The patient received a diagnosis of PCA and started cognitive rehabilitation. The treatment initially led to a slight improvement of all impaired functions except for language skills –in fact, previously unobserved signs of anomia appeared at the first control evaluation, 8 months after diagnosis. However, since 19 months after diagnosis, a gradual decline became evident in visuospatial and visuoperceptual functions and visual attention, accompanied by worsening anomia and semantic dysfluency. Memory skills, though initially unimpaired, also became compromised 21 months after diagnosis. Disturbances of language, memory, and, most notably, visuospatial abilities progressed over time. By the age of 71, the patient presented severe cognitive deterioration, with very low performance in general screening tasks. For a more detailed description of the patient’s cognitive profiles at different stages, see Esteves et al. (2018).

## Materials and Methods

### MRI Analysis

#### Control MRI Sample

We obtained MRI scans from 16 healthy male controls, with a mean age of 70.19 (*SD* = 5.28) and an average of 17.25 years of education (*SD* = 2.11). This sample size was established by reference to previous single-case studies yielding robust results with control groups of 13–16 subjects (Gorno-Tempini et al., 2004; Migliaccio et al., 2012; Spinelli et al., 2015). Control participants had no history of neurological or psychiatric conditions. As shown by Crawford’s modified *t*-tests (Crawford and Garthwaite, 2002, 2012; Crawford et al., 2011), this sample was matched with the patient for age (Eval-1: *t* = 0.15, *p* = 0.88, two-tailed probability; Eval-2: *t* = 0.52, *p* = 0.61, two-tailed probability) and education (*t* = -0.11, *p* = 0.91, two-tailed probability). Note that Crawford’s *t*-test is robust for non-normal distributions, presents low values of type I error, and has yielded replicable results in previous single-case studies –even when individual scores are compared with data from small samples (Baez et al., 2013; Couto et al., 2013; García et al., 2016).

The study was carried out in accordance with the recommendations of the Ethics Committee of the Institute of Cognitive Neurology (INECO, now a host institution of the Institute of Cognitive and Translational Neuroscience). All subjects gave written informed consent in accordance with the Declaration of Helsinki, and written informed consent was obtained from the patient for the publication of this case report. The protocol was approved by the Ethics Committee of INECO. Demographical and MRI data from the patient and controls are available upon request.

#### Image Acquisition

MRI acquisition and pre-processing steps are reported following the practical guide from the Organization for Human Brain Mapping (Nichols et al., 2017; Poldrack et al., 2017). The patient and the controls were scanned in a 1.5-T Philips Intera scanner with a standard head coil (eight channels). We used a T1-weighted anatomical 3D spin echo sequence that covered the whole brain. Structural T1 scans were acquired parallel to the plane connecting the anterior and posterior commissures with the following parameters: matrix size = 256 × 224 × 256 (for Eval-1) and 175 × 256 × 256 (for Eval-2), 1 mm isotropic, repetition time (*TR*) = 7489 ms, echo time (*TE*) = 3420 ms, flip angle = 8°, and sequence duration = 7 min.

#### Voxel-Based Morphometry Pre-processing

VBM was performed to account for the patient’s global atrophy pattern in Eval-1 and Eval-2. Structural images from the patient and the control group were pre-processed with the DARTEL Toolbox from Statistical Parametric Mapping software (SPM12),^1^ following previous procedures (Ashburner and Friston, 2000; García-Cordero et al., 2016; Melloni et al., 2016; Sedeño et al., 2017). T1-weighted images in native space were segmented in gray matter, white matter, and cerebrospinal fluid volumes, using the default parameters of the SPM12 (bias regularization was set to 0.001 and bias FWHM was set to 60 mm cut-off). The following step consisted in applying the DARTEL (create templates) module, with the default parameters of SPM12, to create a template of gray matter that is generated from the complete data set –thus increasing the accuracy of inter-subject alignment (Ashburner, 2007). Then, we ran the “Normalize to MNI Space” module to affine register the last template and the individual gray matter segmented maps, from the previous step, into the MNI space. Subsequently, all images were modulated to correct volume changes by Jacobian determinants, and to avoid bias in the intensity of an area due to its expansion during warping. Then, a 12 mm full-width half-maximum kernel was applied to all images, based on previous recommendations (Ashburner and Friston, 2000; Good et al., 2001). This step leads the data to present a more normal distribution for subsequent parametric analysis. Finally, total gray matter volume was obtained via the VBM8 toolbox for SPM12^2^ and used as a nuisance covariate in the image statistical analysis.

### Semantic Fluency Tasks

In both Eval-1 and Eval-2, the patient’s word retrieval skills were assessed via semantic fluency tasks (administered in Spanish, the patient’s native language). In the noun condition, the patient was given 1 min to name as many animals as possible. Then, in the action-verb condition, the patient was allotted another minute to mention as many actions as he could. The patient was instructed not to repeat words and, when he did, these were excluded from the analyses. Words belonging to the same lemma, but with a different derivational suffix [e.g., *perro* (*dog*), *perra* (*female dog*), *perrito* (*small dog*)] or hyperonyms [e.g., *paloma* (*dove*), *ave* (*bird*)] were also rejected. In the action-verb task, only infinitives were accepted. During the test, the examiner remained quiet and no clues were provided. Answers were given orally, audio-recorded, and then transcribed.

### Statistical Analysis

#### Image Statistical Analysis

To establish the patient’s whole-brain atrophy pattern in Eval-1 and Eval-2, we compared his pre-processed imaged with those of the control group via *t*-tests in a second-level analysis within general linear models on SPM12, following previous studies (Baez et al., 2013; García et al., 2016). Only voxels with a gray matter intensity value greater than 0.2 (maximum value = 1) were entered to avoid edge effects around the border between gray matter, white matter, and cerebrospinal fluid (Friederich et al., 2012). We included total gray matter volume as a covariate in the group comparison to exclusively identify regional changes that are not explained by global effects (Friederich et al., 2012). In line with formal recommendations (Poldrack et al., 2008) and previous reports (Whitwell et al., 2007b; Zamboni et al., 2008), the statistical threshold was set at *p* < 0.001 uncorrected, with an extension of 30 voxels.

#### Fluency Performance Analysis

The patient’s fluency performance was compared to validated age- and educational-level-matched norms for the Argentine population (animals: *n* = 63, *M* = 19.5, *SD* = 5.5; verbs: *n* = 25, *M* = 21, *SD* = 5.73) –for further data on the sample yielding these normative values, see (Butman et al., 2000; Abraham et al., 2008). The results were compared through Crawford’s *t*-tests (Crawford and Howell, 1998; Crawford and Garthwaite, 2002, 2012; Crawford et al., 2009, 2011). As stated above, this test provides robust information for non-normal distributions, presents low rates of type-I error, and has yielded robust results in previous single-case studies (Straube et al., 2010; Couto et al., 2013, 2014; García et al., 2016, 2017c).

## Results

### Eval-1 Results

#### VBM Results

**FIGURE 1:**
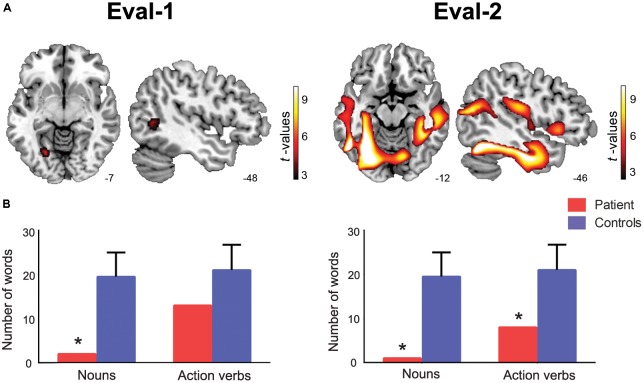
Anatomical and behavioral results. **(A)** Patient’s atrophy pattern at the initial evaluation (Eval-1, age 71, left panel) and the second evaluation (Eval-2, age 73, right panel). The image evidences the progression of neurodegeneration from posterior regions (in Eval-1) to frontal and motor regions (in Eval-2), as compared to healthy sociodemographically matched controls (*p* < 0.001 uncorrected, with an extension of 30 voxels; coordinates are in MNI space). **(B)** Patient’s fluency performance compared to age-matched norms. Noun fluency was affected in both assessments, whereas action-verb fluency proved impaired only in Eval-2. The asterisk (^∗^) indicates significant differences (*p* < 0.05 and *p* < 0.001) between the patient’s performance and normative values.

Relative to controls, the patient exhibited atrophy in left posterior regions, namely, the lingual and middle temporal gyri (for details, see **Table 1** and **Figure 1A**).

**Table 1:** Patient’s atrophied areas in the initial evaluation.

Cluster	Peak *t*	MNI coordinates			Regions
Number of voxels		*x*	*y*	*Z*	
30	4.89	-48	-57	3	Left middle temporal gyrus
34	4.26	-22.5	-66	-6	Left lingual gyrus

#### Fluency Results

The patient’s semantic fluency performance on Eval-1 revealed a dissociation between noun and action-verb processing (**Figure 1B**). On the one hand, he retrieved only two words in the noun condition, thus exhibiting a significant impairment relative to age-matched norms (*Z* = -3.2; *t* = -3.2; *p* = 0.002). On the other hand, he produced 13 action verbs, which indicated low but normal performance in verb fluency compared to controls (*Z* = -1.4; *t* = -1.4; *p* = 0.18).

### Eval-2 Results

#### VBM Results

In Eval-2, the patient’s atrophy progressed from posterior to central and frontal regions, including the thalamus, the cerebellum, the rolandic operculum, the inferior and superior frontal gyrus, and the supramarginal gyrus (for detailed areas of atrophy, see **Table 2** and **Figure 1A**).

**Table 2:** Patient’s atrophied areas in the second evaluation.

Cluster	Peak *t*	MNI coordinates			Regions
Number of voxels		*x*	*y*	*Z*	
31803	28.53	-51	-61.5	9	Left middle temporal gyrus
1542	8.99	-46.5	-21	21	Left rolandic operculum
741	7.79	-49.5	16.5	-3	Left inferior frontal gyrus, triangular part
99	5.48	-24	-43.5	-43.5	Left cerebellum
195	5.13	-13.5	-19.5	12	Left thalamus
48	4.61	-21	48	39	Left superior dorsolateral frontal gyrus
91	4.09	51	-45	28.5	Right supramarginal gyrus

#### Fluency Results

Fluency assessment at Eval-2 revealed significant deficits in both categories (**Figure 1B**). Specifically, the patient was capable of naming only one animal (*Z* = -3.4; *t* = -3.3; *p* = 0.001) and eight action verbs (*Z* = -2.3; *t* = -2.2; *p* = 0.036).

## Discussion

The present study aimed to assess the dissociation between noun and action-verb production in a longitudinal lesion model showing earlier atrophy of posterior regions (Eval-1) and later compromise of fronto-motor regions (Eval-2). Results showed that, in Eval-1, the patient evinced a selective deficit in noun fluency, whereas action-verb difficulties became significant only in Eval-2, supporting the critical reliance of action verbs on (embodied) motor mechanisms. These findings are discussed below.

In Eval-1 (age 71), the patient’s selective atrophy of left posterior regions (middle temporal and lingual gyri) was accompanied by impaired noun retrieval but spared action-verb production. Compatibly, previous research has repeatedly shown marked noun impairments in aphasic patients with predominant left temporal and occipital lesions (Luzzatti et al., 2006), even when verb processing was spared (Damasio and Tranel, 1993). In line with these findings, our Eval-1 results suggest that when neural damage spares anterior/motor regions, processing of action verbs is not significantly compromised.

In Eval-2 (age 73), when atrophy had extended to the supramarginal gyrus and, more crucially, to various regions subserving motor function –namely, the thalamus (Sommer, 2003), the cerebellum (Manto et al., 2012), the rolandic operculum (Brown et al., 2007, 2009), and the inferior and superior frontal gyri (Dassonville et al., 2001; Gardini et al., 2016)–, the patient’s noun fluency deficits were accompanied by action-verb difficulties. This aligns with evidence that left posterior frontal lesions typically involve greater impairments for verbs than nouns in naming and repetition tasks –even with ambiguous items and pseudo-words– (Shapiro and Caramazza, 2003), and that action verbs may become selectively compromised in patients with frontostriatal motor network atrophy (Birba et al., 2017; Bocanegra et al., 2017; García et al., 2017a). The patient’s anatomo-clinical profile in Eval-2 extends such findings, further suggesting that action-verb deficits are distinctly related to damage of anterior/motor regions, as opposed to other portions of the vast language network.

Despite the converging evidence described above, other approaches have yielded contradictory results. For example, difficulties in regular (but not irregular) verb production have been reported in a subset of stroke patients with left ventral supramarginal damage (Meteyard et al., 2013). Also, left inferior frontal degeneration in non-fluent primary progressive aphasia has been associated with deficits in both verb and noun production (Marcotte et al., 2014). Although, *prima facie*, these results would seem to jeopardize the dissociation postulated above, such conflicting findings may reflect methodological limitations, such as the heterogeneous nature (Luzzatti et al., 2002; Mätzig et al., 2009) and anatomical inconsistency (Berndt et al., 1997; Thompson et al., 2012) of regions compromised across patients with focal damage, as well as the diffuse nature of neurodegenerative disorders (Bird et al., 2000; Cousins and Grossman, 2017; Shebani et al., 2017). Moreover, many of the studies reporting trans-categorical deficits lacked anatomical mappings of lesion extent (De Renzi and Di Pellegrino, 1995; Berndt et al., 1997; Luzzatti et al., 2002; Mätzig et al., 2009; Bocanegra et al., 2017; García et al., 2017c), so they cannot firmly rule out the dissociation postulated herein; indeed, exclusively clinical confirmation of a disorder is blind to the actual advance of physiopathology.

Importantly, too, noun and action-verb deficits are differentially related to overall cognitive status: when domain-general functions are compromised, nouns are impaired as much as verbs, even in disorders not characterized by posterior damage (Birba et al., 2017; Bocanegra et al., 2017; García et al., 2017a). Given that most studies failing to replicate this double dissociation did not assess the role of domain-general functions in the patients’ performance, noun deficits in fronto-motor disorders may have emerged as an epiphenomenon to overall (non-language-specific) alterations. Moreover, note that several studies have looked at verbs in general (Miceli et al., 1988; Zingeser and Berndt, 1990; Cappa et al., 1998; Kim and Thompson, 2000; Luzzatti et al., 2006; Tyler et al., 2008; Silveri et al., 2017), which are widely varied in their psycholinguistic properties and depend on widely distributed networks covering multiple brain regions (Marshall et al., 1998; Bird et al., 2000; Shapiro et al., 2006). Thus, at least in some of these studies, verb deficits in patients with posterior lesions may reflect difficulties in lexical subsets whose putative bases are more widely distributed across posterior lobes.

Considering the above caveats, our study represents a useful complementary approach to assess the neurofunctional organization of action verbs and nouns. First, note that PCA is characterized by posterior-to-anterior atrophy, and that nouns are easier to process than verbs in terms of argumental structure (Thompson, 2003), syntactic complexity (Goodglass and Geschwind, 1976; Zingeser and Berndt, 1990; Berndt et al., 1997), morphology (Tyler et al., 2004), and imageability (Crepaldi et al., 2006). Thus, our longitudinal approach created a stringent testing ground for our hypothesis, since a selective deficit for nouns in Eval-1 (when atrophy was confined to posterior regions) could not be explained by coarse category-related demands. Second, using VBM, we confirmed the location and extent of atrophy in each time point, which enabled us to move beyond the neural uncertainty of neuropsychological studies based solely on clinical evidence. Third, by employing a longitudinal design focused on a progressive, single-subject atrophy model, we circumvented confounds related to within-sample heterogeneity in multi-participant studies on language disorders (Nespoulous, 2000; Krishnan et al., 2012).

More generally, our study meets the requisite of incorporating single-subject studies in cognitive neuroscience, as a crucial complement of group-based neuroimaging research (Dubois and Adolphs, 2016). As shown by previous works, establishing particular patterns of compromised and spared functions given circumscribed MRI/fMRI abnormalities in theoretically relevant single cases is highly useful for tracking particular forms of functional organization, impairment, and re-organization (Baez et al., 2013; Sedeño et al., 2014; Couto et al., 2015; García et al., 2017a,c; Ibáñez et al., 2018). A dialog between individual-level findings and group-based outcomes is critical to foster progress in both theoretical and applied areas of the field.

In this sense, the dissociation evinced through our design is also supported by research on healthy subjects. Evidence from fMRI studies in multiple languages (e.g., English, German, Spanish, Italian) has repeatedly shown increased activation in frontal/pre-frontal regions for verbs and middle/posterior temporal regions for nouns (Martin et al., 1995; Koenig and Lehmann, 1996; Perani et al., 1999; Shapiro et al., 2006). Moreover, EEG research has revealed increased early frontal negativity modulations for verbs compared to nouns (Brown et al., 1999) and enhanced N400 amplitudes –a component with sources in temporal regions (Lau et al., 2008; Kutas and Federmeier, 2011)– for nouns compared to verbs (Lee and Federmeier, 2006). In addition, transcranial magnetic stimulation of the primary motor cortex systematically yields selective effects for verbs, in general (Shapiro et al., 2001; Cappelletti et al., 2008), and action verbs, in particular (Gerfo et al., 2008; Tomasino et al., 2008; Liuzzi et al., 2010; Innocenti et al., 2014). Our study extends these single-time-point studies with compatible longitudinal evidence.

From a theoretical stance, the present findings can constrain models of lexico-semantic organization in the brain. Nouns (in this case, names of animals) jointly evoke visual, auditory, somatosensory, and higher-level properties which are crucially subserved by convergence hubs in temporal regions (Patterson et al., 2007; Binder and Desai, 2011; Seghier, 2013; Ralph et al., 2017). On the other hand, action verbs are characterized by denoting patterns of bodily movement, whose learning, execution, and control rely on motor regions, including those affected in the patient (Bak, 2013; Bocanegra et al., 2015; García et al., 2016; Abrevaya et al., 2017; Birba et al., 2017). Rather than mere anatomo-functional coincidences, these associations likely reflect the embodied foundations of language mechanisms. From this perspective, the neural regions engaged for the processing of specific word categories are largely driven by the sensorimotor experiences they allude to (Pulvermüller, 2005; García and Ibáñez, 2014, 2016a,b; Moseley and Pulvermüller, 2014; Zwaan, 2014; Horoufchin et al., 2018).

In this sense, a key theoretical contribution of our work is that the successive compromise of experientially specialized networks is mirrored by the temporal ordering of lexico-semantic alterations. Whereas the embodied framework has incorporated a diachronic dimension thanks to relevant studies on child development (Glenberg et al., 2011; Wellsby and Pexman, 2014; Marshall, 2016), such a perspective is missing from models of adult embodied cognition. Given that sustained motor activity is causally related to action-language comprehension (e.g., Trevisan et al., 2017), reduced motility in the elderly could be expected to decrease the reliance of action-verb processing on putative motor networks. Our findings suggest that this is not the case, even when less psycholinguistically accessible word classes are significantly affected. Insights along these lines, supported by longitudinal lesion models, could offer important constraints to consolidate age-sensitive accounts of other embodied language processes, such as categorization (Dalla Volta et al., 2018) and syntactic processing (Casado et al., 2018).

Moreover, although lesion-based embodied models of action-verb processing have mainly drawn on movement disorders (Cardona et al., 2013; Birba et al., 2017) and asymptomatic samples at risk for such pathologies (Kargieman et al., 2014; García et al., 2017b) our study indicates that important insights can be gained by examining non-motor diseases involving specific patterns of motor network sparing and damage. In this sense, the neurofunctional segregation of circuits subserving action-verb and noun association has been informed by evidence from frontal variant frontotemporal dementia (Bak and Hodges, 2003), a non-motor disease secondarily compromising motor circuitry. Further explorations in relevant patient samples, even if not primarily characterized by movement alterations, could expand the basis for empirico-theoretical developments in the field.

In addition, our findings bear clinical implications. A recent translational proposal, known as the “disrupted motor grounding hypothesis” (Birba et al., 2017), underscores the relevance of action-verb deficits as potential sensitive biomarkers of motor-network atrophy, including anterior/motor and frontostriatal regions. Importantly, validation of this proposal requires accruing evidence that such deficits do not emerge when movement-related regions are spared, particularly if other word categories are affected. Our study shows precisely that: in Eval-1, action-verb production was preserved, despite significant posterior atrophy and accompanying noun fluency deficits. By corroborating this progressive anatomo-clinical link at the *single-patient level* (namely, the level at which clinical interventions operate), our study refines the group-based rationale supporting the disrupted motor grounding hypothesis, addressing recent calls for personalized investigations of brain function (Dubois and Adolphs, 2016).

## Limitations and Avenues for Further Research

This work has some limitations. First, the MRI control sample had a moderate size. Note, however, that robust and replicable results have been obtained in previous reports employing similar or smaller control sample sizes (Crepaldi et al., 2006; García et al., 2016; Shebani et al., 2017), or even no control group at all (Damasio and Tranel, 1993; Shapiro and Caramazza, 2003). Also, our use of the same control sample for both time points may have partially amplified VBM differences in Eval-2. Although the same strategy has been successfully employed in previous research (Gorno-Tempini et al., 2004; Chan et al., 2015), future studies should examine whether our results are replicated when using a control sample specifically matched on a one-to-one basis to the patient’s age in each time point. Third, the independence of neuroanatomical and behavioral results in the control sample may have impacted our results. It would thus be useful to replicate our study with behavioral outcomes from the control sample as opposed to normative data. Fourth, despite their relevance and wide use, fluency tasks allow for no control over the psycholinguistic properties of words in each condition. Future studies should aim to extend this work with additional tasks, including not only productive but also receptive paradigms. It might also be argued that the animal category has a lesser productivity than the action category, which may have influenced the differential pattern observed in Eval-1. Yet, even if this were the case, this could hardly account for the specific pattern of deficits we detected, as such a difference was held constant for both the patient and controls. Finally, it would be interesting for future studies to replicate our single-case approach including other lexical categories, more particularly, different classes of action and non-action verbs.

## Conclusion

This longitudinal single-case study shows that while action verbs, as opposed to nouns, can be spared in the context of temporal brain atrophy, they become significantly compromised when neurodegeneration reaches frontal and subcortical motor hubs. Our progressive lesion-model approach constitutes an informative complement to more traditional one-time group- and patient-level research on the neural organization of lexical categories. Future studies along these lines could further our understanding of the functional organization of language, while opening new windows for translational extensions of the embodied cognition framework (García and Ibáñez, 2018).

## Author Contributions

JF, MC, AI, and AMG conceived and designed the study. GB evaluated the patient and administered the fluency tests. IG-C, LS, and LC analyzed the images. MC performed the statistical analysis of the fluency tests. IG-C, LS, and AMG designed **Figure 1**. BS, MCH, IG-C, and AMG wrote the manuscript. AI and LS provided critical revisions on successive drafts. All authors approved the manuscript in its final form.

## Conflict of Interest Statement

The authors declare that the research was conducted in the absence of any commercial or financial relationships that could be construed as a potential conflict of interest.

## Footnotes

1 http://www.fil.ion.ucl.ac.uk/spm/software/spm12/

2 http://www.neuro.uni-jena.de/vbm/
